# Nosocomial Dengue by Mucocutaneous Transmission

**DOI:** 10.3201/eid1105.040934

**Published:** 2005-05

**Authors:** Lin H. Chen, Mary E. Wilson

**Affiliations:** * Mount Auburn Hospital, Cambridge, Massachusetts, USA;; † Harvard Medical School, Boston, Massachusetts, USA

**Keywords:** dengue, nosocomial, mucocutaneous, blood-borne pathogen, travel illness, virus transmission

**To the Editor:** Wagner and colleagues report nosocomial dengue transmitted by needlestick and note that it is the fourth case of nosocomial dengue to their knowledge (1). In the same issue of Emerging Infectious Diseases, Nemes and colleagues report a separate case of nosocomial dengue also transmitted by needlestick (2). Three other cases of nosocomial dengue transmission by needlestick have previously been published (3–5).

We have recently published a case of nosocomial dengue infection that was transmitted by mucocutaneous exposure to blood from a febrile traveler who had recently returned from Peru (6). During phlebotomy, a healthcare worker was splashed in the face with the traveler's blood. Both the traveler and the healthcare worker were subsequently found to have dengue fever with dengue virus type 3. This route of infection is biologically plausible because infection through mucosal surfaces (intranasal and oral routes) has been shown possible for arboviruses (7). In our review of the literature, we also found a report of dengue virus transmission by bone marrow transplantation (8). Other cases of transmission of dengue virus without a mosquito vector have occurred in 5 reported instances of infection in newborns as a result of intrapartum or vertical transmission from mother to child (9–12).

We agree that nosocomial transmission may become more common in temperate areas as more travelers return home with acute dengue fever. As Wagner and colleagues pointed out, travelers visiting Southeast Asia have the greatest risk of acquiring dengue infections because of the high endemicity of these viruses there. Our report further illustrates the occurrence of dengue infection in the Americas (13) and the risk for dengue to travelers visiting this region. Among 33 returned travelers with dengue infection reported in the United States in 1999 and 2000, 20 had acquired infection in the Caribbean islands (12 cases) or Central or South America (8 cases) (14). Clinicians and laboratorians should be alert to the possibility of acquiring infection with a dengue virus after needlestick or mucocutaneous blood exposure. The magnitude of nosocomial transmission in dengue-endemic areas is unknown and more difficult to assess because healthcare workers may be exposed to dengue virus–infected mosquitoes outside the clinical setting.
